# Faciobrachial Stroke as the Initial Presentation of Moyamoya in a Pediatric Patient Precipitated by Mycoplasma pneumoniae Pneumonia: A Case Report and Comprehensive Review of the Literature

**DOI:** 10.7759/cureus.81979

**Published:** 2025-04-09

**Authors:** Sara S Dhanawade, Prajakta S Ghatage

**Affiliations:** 1 Pediatrics and Child Health, Bharati Vidyapeeth (Deemed to be University) Medical College and Hospital, Sangli, Sangli, IND; 2 Pediatric Neurology, Bharati Vidyapeeth (Deemed to be University) Medical College and Hospital, Sangli, Sangli, IND

**Keywords:** facio-brachial paresis, monoparesis, moyamoya, mycoplasma pneumoniae pneumonia, stroke

## Abstract

Moyamoya disease is characterized by progressive stenosis of the distal end of the internal carotid arteries with the formation of extensive collaterals. Moyamoya is a common cause of pediatric stroke, but data regarding pediatric-specific etio-clinical profiles and topological co-relation are sparse.

We present a rare case of a 10-year-old girl with left faciobrachial monoparesis due to an acute ischemic stroke in the right subcortical frontoparietal region, with angiography revealing bilateral moyamoya disease. This unique hemodynamic stroke of moyamoya was precipitated by *Mycoplasma pneumoniae* pneumonia.

## Introduction

Moyamoya disease is an important cause of pediatric ischemic stroke. Idiopathic moyamoya is characterized by chronic progressive stenosis or occlusion of the terminal internal carotid arteries (ICAs) and/or the proximal portion of the anterior cerebral arteries (ACAs) and/or middle cerebral arteries (MCA) [1]. Suzuki and Takaku [2] named it moyamoya, which describes the puff of smoke-like appearance of the newly formed collaterals. The current definition of moyamoya is progressive ICA occlusion with compensatory basal cerebral collateral development [3].

Its incidence is 0.54 per 100,000 patients globally. The most common clinical presentation is stroke, followed by transient ischemic attack (TIA), seizure, and headache. Most cases present with unilateral stroke. The most common presentation is an extensive stroke with hemiparesis and facial involvement [4]. Moyamoya presenting with a pure motor stroke with faciobrachial paresis in the pediatric age group is rare.

We report a case of a 10-year-old girl with left faciobrachial palsy due to acute ischemic stroke in the right subcortical frontoparietal region, with angiography showing bilateral moyamoya disease.

## Case presentation

A 10-year-old girl, the first child of a nonconsanguineous marriage, was admitted to our hospital with complaints of fever and cough for one week. Three hours prior to admission, weakness of the left upper limb with a deviation of the face to the right side was noticed. The weakness of the left upper limb was acute, maximum at onset, affecting the distal more than the proximal part. Parents also noted a sudden right-side facial deviation. There was no history of involvement of the right upper limb or both lower limbs, altered sensorium, seizures, or other cranial nerve involvement. There is no relevant birth history. Development was age-appropriate, with no notable prior history. There was no history suggestive of transient ischemic attacks, headaches, or tuberculosis contact.

On admission, the child was vitally stable. Higher functions were found to be normal upon neurological testing, but cranial nerve examination revealed left upper motor neuron (UMN)-type facial palsy. On motor system examination, there was reduced tone in the left upper limb with a power of 1/5, while the power in the other limbs was 5/5. The left upper limb had brisk deep tendon reflexes, while the other limbs showed normal reflexes. Both plantar were flexors.

On auscultation of the chest, air entry was reduced over the left infra-scapular area with crepitations. There was no other positive finding on examination.

Hematological testing revealed leukocytosis with raised C-reactive protein. The patient’s hematological values have been tabulated with reference ranges (Table 1).

**Table 1:** Hematological values with reference range.

Laboratory test	Patient’s lab values	Reference range
Hemoglobin (g/dL)	11.4	12-14
White blood cell count (/cu mm)	13,800	5,000-11,000
Platelet count (/cu mm)	2,48,000	1.5-4,00,000
C-reactive protein (mg/dL)	159	<6
Serum calcium (mg/dL)	8.4	8.4-10.5
Blood urea (mg/dL)	15	12-45
Serum creatinine (mmol/L)	0.5	0.7-1.4
Serum sodium (mmol/L)	140	135-145
Serum potassium (mmol/L)	3.7	3.5-5
Prothrombin time (seconds)	13	11.6
Activated partial thromboplastin time (seconds)	26	26.6
International normalized ratio	1.1	<1.1

**Figure 1:**
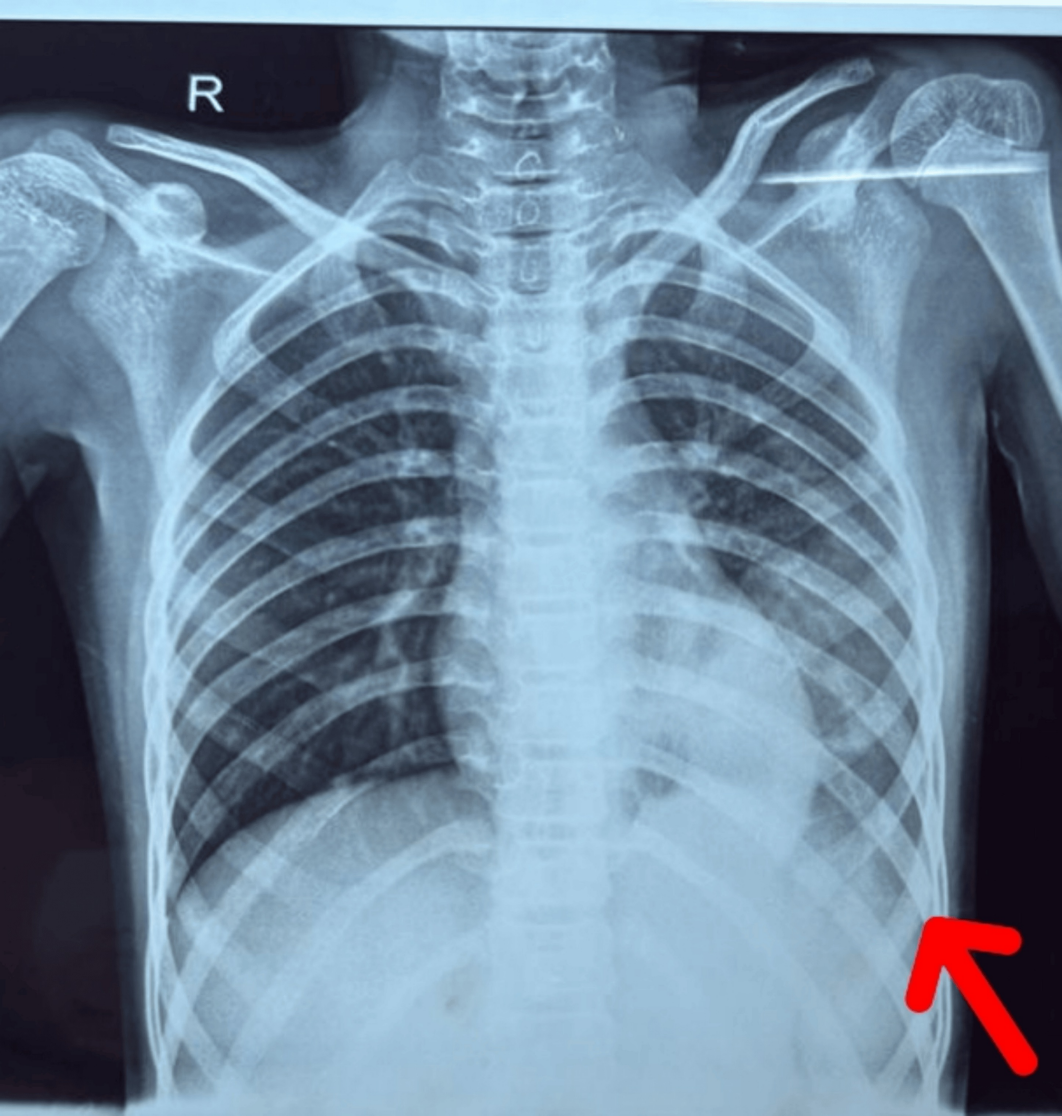
X-ray chest showing left lower lobe consolidation.

X-ray of chest showed radiopacity in the left lower zone, retrocardiac region silhouetting the left hemidiaphragm, features suggestive of left lower lobe collapse consolidation (Figure 1). The *Mycoplasma pneumoniae* IgM antibody was positive at a titer of >27 NTU (reference value <9 NTU). The CSF examination revealed that the protein level was 20.6 mg/dL (20-40 mg/dL), the sugar level was 67 mg/dL (with a corresponding blood sugar of 117 mg/dL), and the white cell count was 5 with 100% lymphocytes, which were all within normal limits. The Gram stain showed no organism. CSF culture revealed no growth.

The thrombotic evaluation yielded normal results (Table 2). Anticardiolipin antibody, factor V Leiden, and lupus anticoagulant were negative. The two-dimensional echocardiogram (2D echo) was normal.

**Table 2:** Laboratory values for thrombotic workup.

Laboratory test	Patient’s lab value	Reference range
Homocysteine (micromol/L)	14.4	5-15
Vitamin B12 (pmol/L)	865	250-1,200
Protein C (%)	110	70-129
Protein S (%)	76	70-120
Antithrombin III (%)	94	80-120

**Figure 2:**
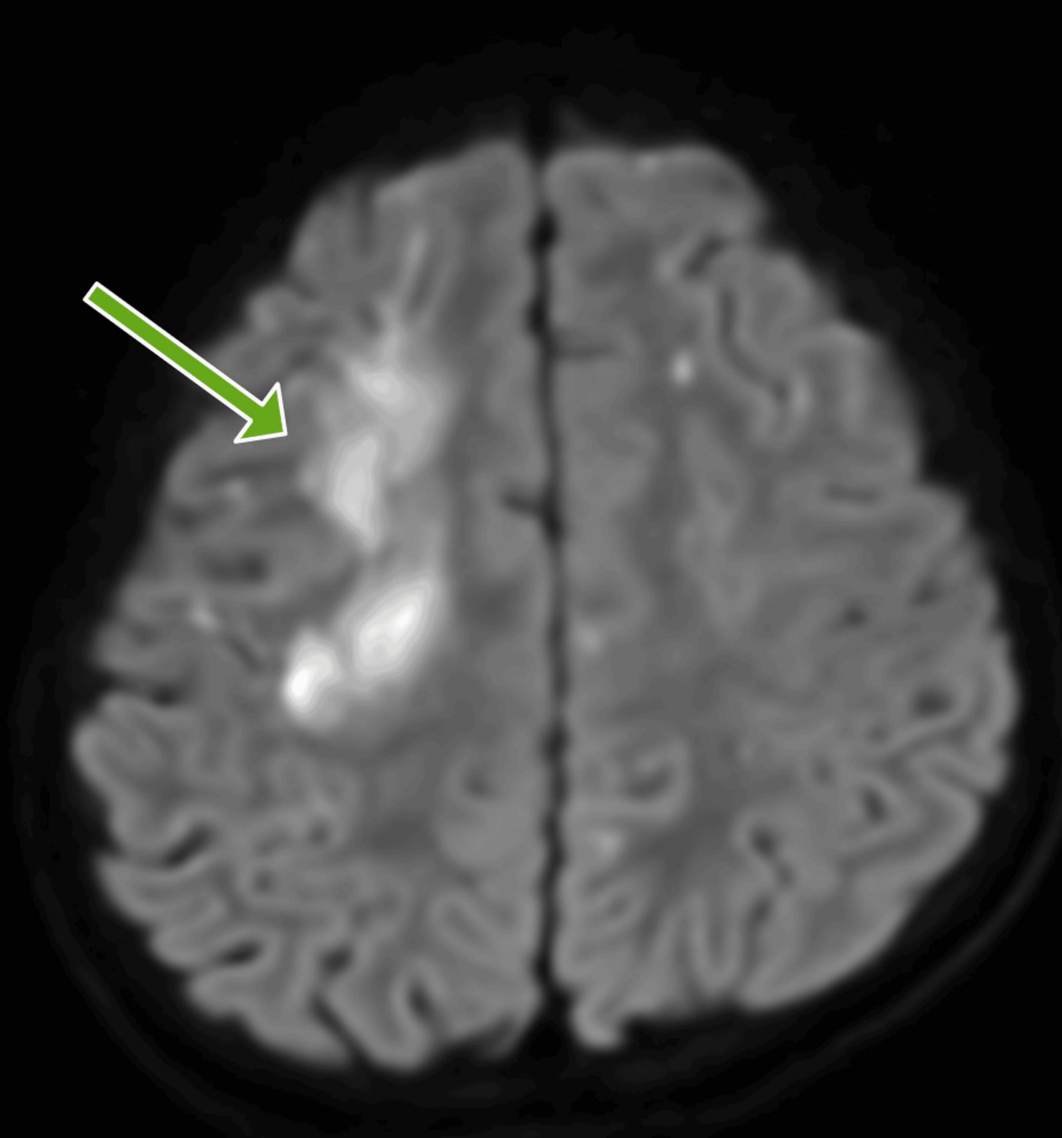
Diffusion-weighted imaging (DWI) showing acute multiple ischemic infarcts in the right frontoparietal area.

**Figure 3:**
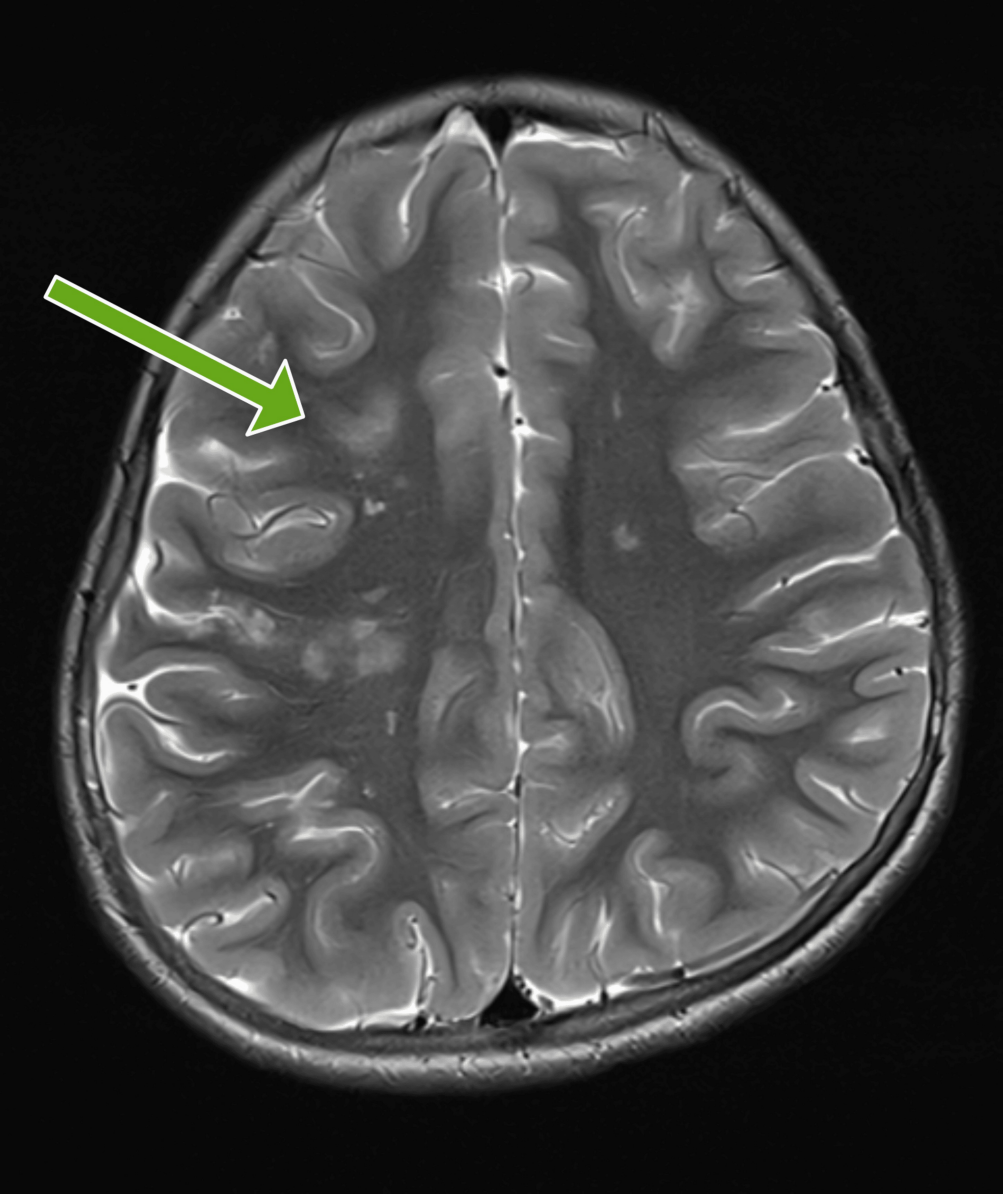
T2-weighted imaging showing hyperintensities in the right frontoparietal region.

MRI brain imaging showed acute multiple ischemic infarcts in the right frontoparietal area and the genu of the corpus callosum. White matter in both the frontoparietal cortical and subcortical regions showed old infarcts (Figures 2, 3). The ischemic infarcts occurred in the watershed area between the ACA and MCA territories.

**Figure 4:**
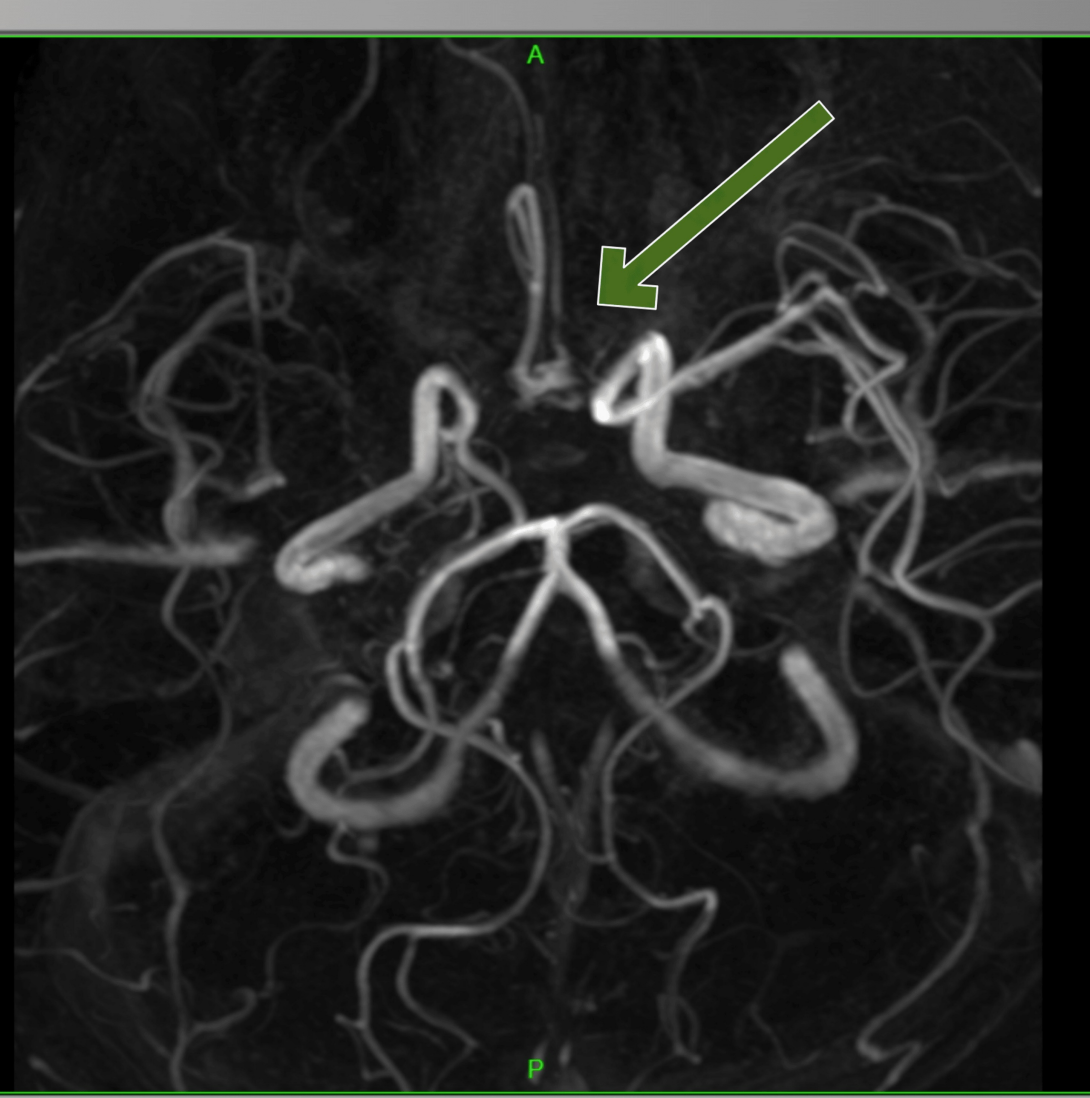
MR angiography showing bilateral anterior cerebral artery (A1) segment, right middle cerebral artery (M1, M2 segment) complete stenosis with extensive collateralization.

**Figure 5:**
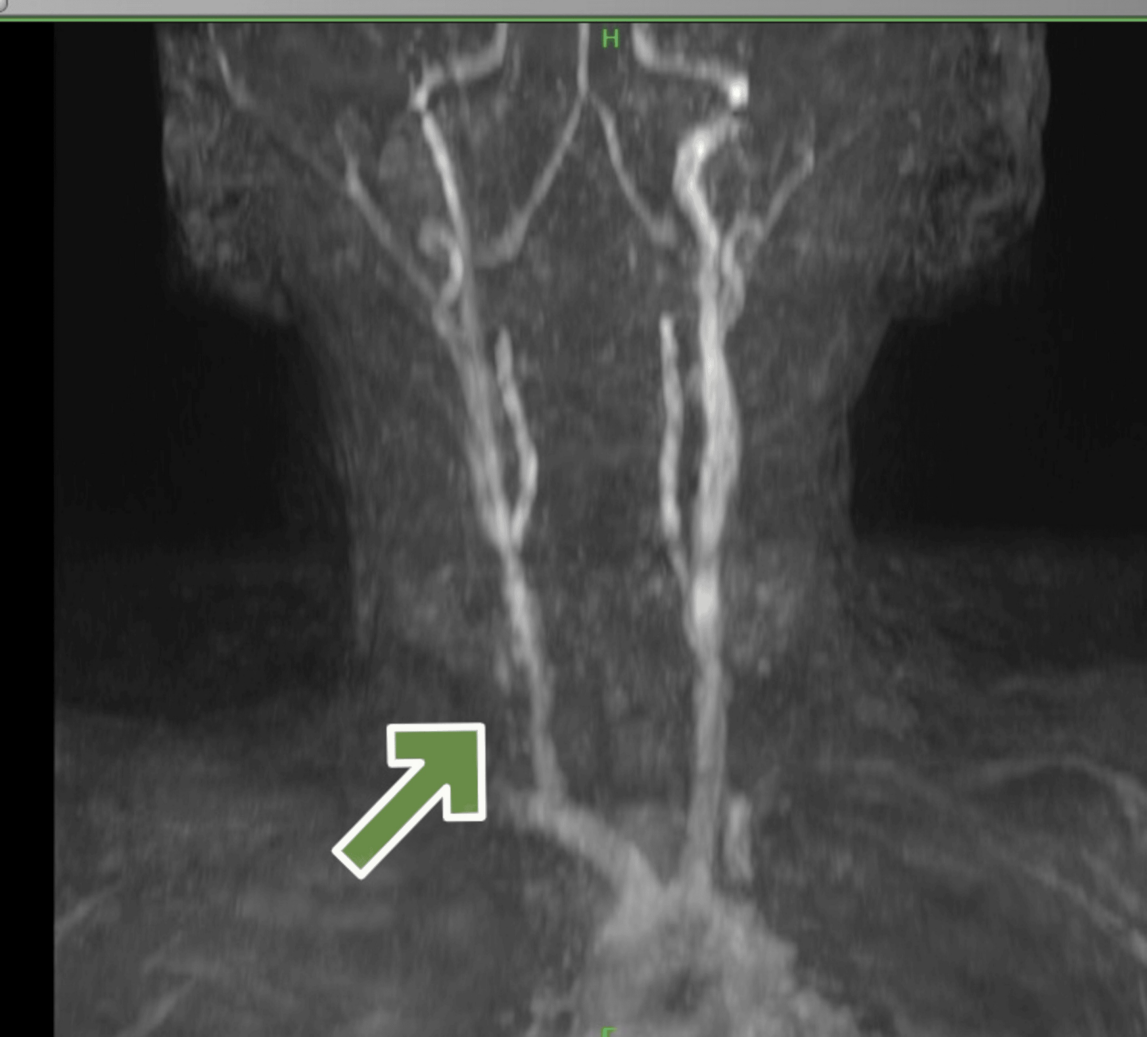
MR angiography showing congenital hypoplastic right internal carotid artery.

MR angiography was suggestive of moyamoya disease with bilateral ACA (A1) segment, right MCA (M1, M2 segment) complete stenosis with extensive collateralization, along with right ICA terminal segment severe stenosis. The right internal carotid had congenital hypoplasia. The left ICA was normal. The A2 segment of the left ACA was hypoplastic, accompanied by compensatory changes in the posterior circulation of the right posterior communicating artery (Figures 4, 5).

She was started on antithrombotic drugs, aspirin (3 mg/kg/day) and low-molecular-weight heparin (1 mg/kg/day). She had improved left upper limb proximal as well as distal power, which was 3/5 over a period of five days. Her pneumonia had completely resolved after treatment with the antibiotics ceftriaxone and azithromycin. Aspirin was maintained in conjunction with physiotherapy. She was also given hematinics for anemia. On follow-up after one month, the power was 5/5 with no new deficits.

## Discussion

Arterial ischemic stroke is a common cause of mortality and morbidity in the pediatric age group. Moyamoya disease, an idiopathic condition, is a significant contributor to childhood stroke, particularly among the Asian demographic [5]. Moyamoya is characterized by progressive obstruction of distal ICAs and branches of the circle of Willis. Compensatory angiogenesis generates collateral vessels to sustain blood supply. Unlike other types of ischemic strokes, moyamoya-associated infarctions are hemodynamic strokes resulting from hypoperfusion. The infarcts are located in the watershed zone as a result of this mechanism.

Additionally, there is a loss of autoregulation in these chronically underperfused arteries, which may lead to stroke being triggered by fluctuations in carbon dioxide levels, such as hyperventilation. In our case, the child had *Mycoplasma pneumoniae* pneumonia, which precipitated the stroke. A prior case report by Alier et al. [6] detailed the case of an adult with community-acquired pneumonia who experienced an ischemic stroke, compounded by underlying sickle cell anemia. Moyamoya disease was diagnosed in the above case after evaluation for stroke.

In 2019, Ramsi et al. [7] described a case of a two-year-old with a massive infarct due to moyamoya disease precipitated by *Mycoplasma pneumoniae* pneumonia, but the child had presented with a seizure as the initial presentation of moyamoya.

The predominant manifestation of moyamoya is stroke or TIA. Other presentations include headaches, movement disorders, and seizures. Approximately 80% of strokes are ischemic. Hemorrhagic strokes are usually seen more commonly in adult patients [8].

There are limited pediatric case reports of stroke manifesting with motor symptoms affecting the face and upper limbs while sparing the lower limbs. This pattern of faciobrachial paresis is attributed to ischemia, mainly in the motor cortex. Nevertheless, there are limited documented instances of faciobrachial palsy with ischemia affecting the genu of the internal capsule, the region of the Heubner artery, and the pons.

In a comprehensive multicenter study conducted by de Freitas et al. [9] encompassing 895 stroke cases, the incidence of motor stroke affecting both upper and lower limbs alongside facial palsy was 72%; facial and ipsilateral upper limb involvement was 23.2%, and isolated facial palsy was 5.3%. There is a paucity of data regarding pediatric facio-brachial paresis incidence.

The motor pattern of faciobrachial monoparesis has been reported in strokes related to MCA occlusion, ACA occlusion, involvement of the genu of the internal capsule, Heubner artery occlusion, and also the involvement of corticospinal tracts in the pons [10]. We have examined the topological localization of faciobrachial paresis in greater detail.

The selective topological involvement of face and upper limb (distal > proximal upper limb) can be elucidated by their proximity in the motor homunculus. The area representing the face and distal upper limb lies in close proximity on the lateral side near the Rolandic area compared to the area with leg representation, which is more medially in the frontal region. This area represents the face and upper limb region and lies in the MCA and ACA territory watershed zone [11].

Border zone or watershed infarcts are ischemic infarcts that are seen at the junction between two arteries and are at a higher risk of infarction. Border zone infarcts account for about 10% of all cerebral ischemic strokes. The external or cortical border zone infarcts are seen in the border zone area between the anterior, middle, and posterior cerebral artery territories. The internal or subcortical border zones are located in the area between the anterior, middle, and posterior cerebral artery territories and the Heubner, lenticulostriate, and anterior choroidal artery territories as described by Torvik [12]. In our case, the infarct appears to be an external border zone between the MCA and ACA.

In our case, acute ischemic infarcts were present in the right frontoparietal area and genu of the corpus callosum, alongside old, numerous infarcts observed in the bilateral frontoparietal cortical and subcortical white matter. MR angiography showed bilateral ACA A1 segment, right MCA M1, M2 segment with complete stenosis with extensive collateralization, along with right ICA terminal segment severe stenosis. In a study by Torvik [12], the vascular territory of the stroke in patients with pure monoparesis was identified as superficial MCA in 48%, subcortical (anterior lenticulostriate) in 31%, brainstem in 8%, and ACA in 8%. In the other individuals, the vascular lesions were distributed over different regions.

A study by Castaldo et al. [13] looked at 35,818 patients with monoparesis over the course of five years. The study only looked at distal arm motor paresis as a clinical sign. The results showed a strong link between arm monoparesis and small cortical lesions in the MCA territory with MRI and diffusion-weighted imaging. Their findings align with our study, which identified the total blockage of MCA M1 and M2.

In our case, there is involvement of both the MCA and ACA arteries on the right side. Most instances of solitary ACA A1 segment occlusion manifest with leg deficits corresponding to the motor homunculus. Nevertheless, limited case studies have documented faciobrachial deficits resulting from ACA occlusion, particularly with a recurrent branch of Heubner, which innervates the posterior segment of the anterior limb of the internal capsule and basal ganglia. Faciobrachial monoparesis is presumed secondary to lesions involving the Heubner artery (a proximal perforating branch from the ACA) or the lateral lenticulostriate artery (a branch of the MCA).

In our case, the child presented with left-sided upper limb monoparesis with left UMN facial palsy. Capsular genu syndrome is described as facial palsy with mild monoparesis of the upper limb, which arises because of the involvement of corticobulbar and corticopontine fibers at the genu of the internal capsule [14]. However, diffusion-weighted brain MRI in this case showed an ischemic infarct in the right frontoparietal region.

Vascular procedures are an alternative to the traditional therapy of moyamoya disease, which mostly consists of antithrombotic medications like aspirin. Vascular surgery is generally recommended as the treatment of choice for patients with recurrent or progressive cerebral ischemic events. Two types of vascular procedures can be done: direct and indirect vascularization. In direct vascularization, the superficial temporal artery is anastomosed with the MCA. This technique is difficult to perform but, when successful, will yield excellent results with reperfusion of blood flow. Multiple techniques of indirect anastomotic procedures have been described: encephaloduroarteriosynangiosis (EDAS), whereby the superficial temporal artery is dissected free over a course of several inches and then sutured to the cut edges of the opened dura; encephalomyosynangiosis (EMS), in which the temporalis muscle is dissected and placed onto the surface of the brain to encourage collateral vessel development; and the combination of both, encephalomyoarteriosynangiosis (EMAS) [15,16].

The outcome was excellent in our case, with regaining power 5/5 in the left upper limb on one-month follow-up. In a study by Maeder-Ingvar et al. [17], similar results were seen: 41% of patients with monoparesis were able to return to their previous activities, while 41% required some assistance, 16% were moderately reliant, and 2% were entirely dependent. One percent of patients succumbed during their stay. The same study revealed a substantial difference in outcomes between monoparesis and more extensive motor deficits, mostly attributable to the beneficial outcomes associated with monoparesis.

## Conclusions

This case underlines the rare presentation of moyamoya disease in the pediatric age group. It emphasizes that faciobrachial monoparesis should be considered one of the initial presentations of stroke due to moyamoya. MRI brain with MR angiography is diagnostic of moyamoya disease. Prompt diagnosis and timely management can improve the outcome following moyamoya stroke.

## Appendices

Hereby attaching a diacom link to access the MRI Brain and MR angiography images.

https://sa.lifetrackmed.com/ris/Guest/ViewerNakedLink?X=8f633d42de0708ae01e8c61264df13d9bbac037bec8bf51bb46e3499c38c405a
